# Industrial perceptions of medicines regulatory harmonization in the East African Community

**DOI:** 10.1371/journal.pone.0218617

**Published:** 2019-06-19

**Authors:** Live Storehagen Dansie, Walter Denis Odoch, Christine Årdal

**Affiliations:** 1 Norwegian Institute of Public Health, Oslo, Norway; 2 East, Central and Southern Africa Health Community, Arusha, Tanzania; IMT Institute for Advanced Studies Lucca, ITALY

## Abstract

**Background:**

Medicines regulatory harmonization has been recommended as one way to improve access to quality-assured medicines in low- and middle-income countries. The rationale is that by lowering barriers to entry more manufacturers will be enticed to enter the market, while the capacity at the national medicines regulatory authorities is strengthened. The African Medicines Regulatory Harmonization Initiative, agreed in 2009, is developing regional platforms with harmonized regulatory procedures for the registration of medicines. The first region to implement medicines regulatory harmonization was the East African Community (EAC). The harmonization was based on the existing EAC Free Trade Agreement, which officially launched the free movement of goods and services in 2010.

**Methods and findings:**

In this study we conducted semi-structured interviews and performed document reviews. The main target group for our interviews was pharmaceutical companies. We interviewed 18 companies, including 64% of the total companies who had experienced the EAC joint product assessment procedure, and two EAC-based national medicines regulatory authorities. We found that generally pharmaceutical companies are supportive of the African-based MRH efforts and appreciative of the progress being achieved. However, many companies are now hesitant to use the joint product assessment procedure until efficiency improvements are made. Common frustrations were the length of time to receive the actual marketing authorization; unexpectedly higher quality standards than national procedures; and challenges in getting all EAC countries to recognize EAC approvals. Smaller, less attractive markets have not yet become more attractive from a corporate perspective, and there is no free trade of pharmaceuticals in the EAC region.

**Conclusions:**

Pharmaceutical companies agree that medicines regulatory harmonization is the way forward. However, regulatory medicines harmonization must actually result in quicker access to the harmonized markets for quality-assured medicines. At this time, improvements are required to the current EAC processes to meet the vision of harmonization.

## Introduction

Insufficient access to essential medicines remains one of the biggest public health challenges in Africa [1]. In 2017 almost three million children under the age of five died in Sub-Saharan Africa, more than any other geographic region [2]. Many of these deaths could have been prevented by access to stronger healthcare systems capable of ensuring access to quality-assured medicines and other services. However, the system and capacity to ensure access to quality-assured medicines vary greatly from country to country. Low- and middle-income countries (LMICs) are often faced with resource constraints within their National Medicines Regulatory Authorities (NMRA), the national bodies mandated to ensure appropriate access to safe and quality-assured medicines [3, 4]. A weakly functioning NMRA can result in delayed assessment of marketing authorization applications, which may ultimately promote the introduction of counterfeit medicines [5]. This applies to not only new medicines representing advances in treatment, but also generic medicines.

Harmonization of regulatory requirements for medicines regulation where NMRAs collaborate and share resources and knowledge is one of the solutions. Harmonization offers the possibility to eliminate duplication of work, as well as strengthen the quality and increase confidence in outcomes of dossier assessments. Indeed, there are several prominent examples to support this argument. Europe pioneered regulatory harmonization and moved towards a single market for pharmaceuticals since the 1980s. Following this, a tripartite alliance of Europe, Japan and the United States, the International Council for Harmonization (ICH), was established in 1990. This led to the development of the Common Technical Document (CTD), a common format for submitting a dossier to obtain marketing authorization for medicines. The CTD has since been adopted by several other countries [6]. The European Medicines Agency was created in 1995, and is operating as a decentralised agency evaluating applications for market authorization and monitoring the safety of both human and veterinary medicines in the European Union (EU) [7]. Besides EU and ICH, several regional medicines regulatory harmonization (MRHs) initiatives have been established across the globe, including the Pan American Network for Drug Regulatory Harmonization in 1999 and the African Medicines Regulatory Harmonization (AMRH) Initiative in 2009 [8, 9].

The AMRH Initiative is a consortium consisting of the New Partnership for Africa’s Development (NEPAD) Agency, the Pan African Parliament (PAP), the Bill & Melinda Gates Foundation, the UK Department for International Development (DFID), the Clinton Health Access Initiative (CHAI), the Joint United Nations Programme on HIV/AIDS (UNAIDS) and the World Health Organization (WHO) [10]. The initiative is being implemented regionally, building on the African Union’s Regional Economic Communities (RECs), aiming to develop regional platforms with harmonized technical guidelines, joint dossier assessments and joint Good Manufacturing Practice (GMP) inspections [11]. The East African Community (EAC) was selected as the first region to implement MRH. The EAC consists of the Republic of Burundi, Kenya, Rwanda, South Sudan, the Republic of Uganda, and the United Republic of Tanzania.

MRH offers many potential advantages for low income countries. Some countries like Burundi, Rwanda, and South Sudan have only recently started medicines regulation themselves. MRH facilitates ongoing capacity-building where assessors receive peer learning and feedback. In addition to capacity-building, MRH should theoretically make less attractive markets more attractive. Small, low income countries like Burundi, Rwanda, and South Sudan struggle to attract pharmaceutical manufacturers to serve their individual markets. The combined population of the EAC is more than 170 million, almost putting it on par with the most populous African country, Nigeria. The EAC groups one lower-middle-income country (Kenya) with five low-income countries. The anticipated result is that by lowering the regulatory barriers and administrative costs, pharmaceutical companies registering a product for Kenya will instead opt to seek marketing authorization for the entire region.

The EAC MRH initiative is anchored in the already existing EAC Free Trade Agreement, more specifically in Chapter 21 (Article 118), which makes provisions for the alignment of drug legislation and practices [12]. The free movement of goods and services was officially launched in 2010, followed by the launch of the EAC MRH in 2012 [13]. The EAC Secretariat is the executive body of the EAC. The stated goals of the EAC MRH are to implement harmonized technical documents, an integrated information management system (IMS) for medicines registration and a quality management system in each EAC member state. In addition, it aimed to create a platform for information sharing on a harmonized medicines registration system and to develop and implement a framework for mutual recognition of regulatory decisions made by other EAC member states’ NMRAs [14]. Technical working groups, led by different EAC member states, were created to work on key regulatory activities. This resulted in the launch of harmonized registration guidelines, the CTD and GMP guidelines, in January 2015, and the first joint assessment of dossier applications took place in October 2015 [15].

**Fig 1:**
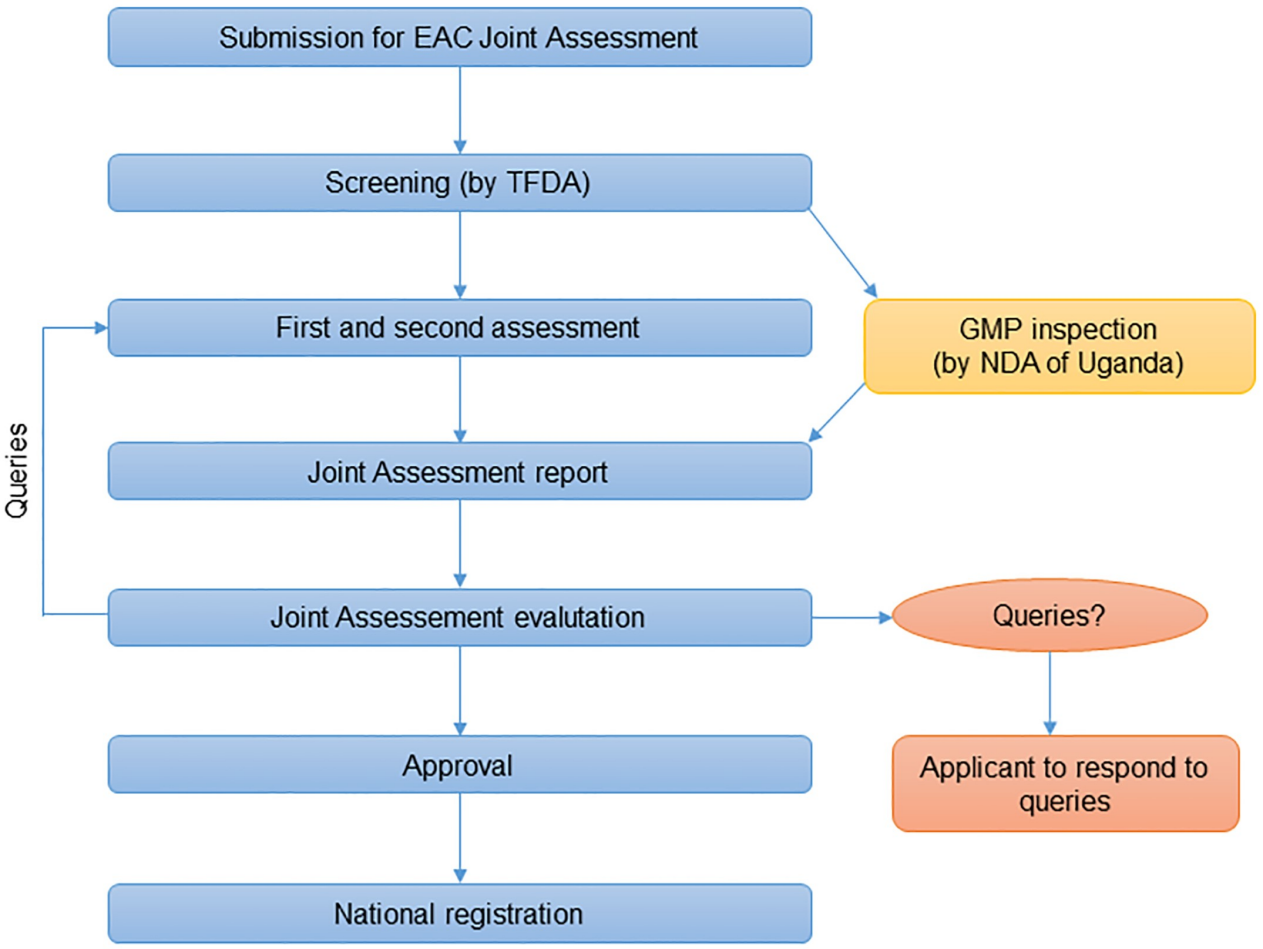
The EAC joint assessment procedure. Source: The EAC procedure for marketing authorization of medicinal products.

The EAC Secretariat established the EAC joint assessment procedure as illustrated in Fig 1. The applicant submits its dossier and related information to Tanzania Food and Drug Authority (TFDA), the lead for joint assessments. TFDA does the first screening. If the dossier is found to be complete, the dossier will be sent to two EAC member states to conduct the first and second assessment, resulting in an assessment report that is circulated to a team of assessors from the EAC member states prior to a joint evaluation meeting. The joint evaluation meetings are conducted by the EAC Secretariat and regional experts on medicines evaluation and registration from the EAC member states, with technical support from the WHO and the Swiss Agency for Therapeutic Products. The National Drug Authority (NDA) in Uganda is the lead for GMP evaluations, which is done prior to the joint assessment evaluation. GMP evaluations ensure that the production facilities meet specified quality standards. Once the team of assessors completes an assessment report, it is sent to the EAC Secretariat who notifies the applicant. In the case of a positive assessment, the EAC NMRAs then have three months from the date of final assessment and joint acceptance to grant national marketing authorization [15]. The EAC Secretariat does not have the formal mandate to grant marketing authorization. Consequently, applicants must submit product applications and pay registration fees to all NMRAs where they seek marketing authorization.

A treaty to establish an African Medicines Agency was recently adopted by the African Union Heads of State and Government [16]. The EAC, being the first region to implement MRH, will act as a point of reference as the remaining RECs implement MRH at the same time as the continent works towards one African Medicines Agency. Therefore, its success can influence the success of forthcoming initiatives. Other regional MRH initiatives have also commenced, including Zazibona (collaboration between NMRAs in Botswana, Namibia, South Africa, Zambia, and Zimbabwe) and in ECOWAS (Economic Community of West African States).

In this study we assess pharmaceutical companies’ perceptions and experiences with the EAC MRH initiative, especially how well it encourages them to serve the EAC market. The results of this study should help to inform the ongoing process of the African MRH efforts.

## Methods

### Ethics

We sought approval for our research portfolio from Kenya’s Kenyatta National Hospital and University of Nairobi Ethics and Research Committee, Tanzania’s National Institute for Medical Research, Uganda’s Higher Degrees, Research and Ethics Committee at Makerere University School of Public Health, and the Norwegian Committees for Medical and Health Research. We received approval from Kenya, Tanzania, and Uganda. The Norwegian Committee decided that our research did not require their ethical approval since we are studying perceptions amongst industrial actors and government employees, not patients. With that said, all interview participants were informed orally that their interview responses would be treated confidentially and that their participation was completely voluntary. Written consent was deemed unnecessary since interview participants responded individually to a call for interviews.

### Study design

In this study we conducted semi-structured interviews and performed document reviews. The main target group for our interviews was pharmaceutical companies. We invited a mix of pharmaceutical companies to participate, including multinational and EAC-based pharmaceutical companies. We identified pharmaceutical companies through industry associations (e.g. the International Federation of Pharmaceutical Manufacturers & Associations (IFPMA) and the Federation of East African Pharmaceutical Manufacturers (FEAPM)). Additional participants were invited to participate through recommendations from interviewees. For the interviews with local manufacturers in Kenya, Tanzania, and Uganda we used local research assistants who helped with the recruitment and scheduling of interviews. We also invited the NMRAs in Kenya, Tanzania, Uganda (the countries where we had sought ethical clearance) and the EAC Secretariat to participate in our study.

Prior to all interviews, an invitation letter explaining the purpose of the interview and the interview guide were shared with the participants. The interviews were conducted at two different time periods. The first interviews were conducted in August-November 2017, and the second set of interviews were conducted in May-June 2018 (after we had received ethical clearances from Kenya, Tanzania, and Uganda). All interviews with local manufacturers were conducted face-to-face, whereas most interviews with multinational companies were conducted over the phone due to their diverse geographical locations world over. The group discussion with multinational companies and industry association representatives took place face-to-face.

### Interviews and participants

We interviewed 18 pharmaceutical companies in total after contacting 22 companies that confirmed receipt of the interview request (response rate of 80%). This was performed through 14 individual interviews and one group interview (with seven multinational pharmaceutical company participants as well as representatives from industry associations) (Table 1). The pharmaceutical companies were a mix of research based (n = 8) and generic (n = 10) companies, and eight manufacturers were based in the EAC. The multinational pharmaceutical companies who participated in both the group interview and an individual interview were with different interviewees. We interviewed seven companies that had experience with the EAC joint assessment procedure, representing 64% of the total companies who had used the procedure until 2017. We interviewed three companies that had experience with the joint GMP inspection, representing 23% of the total companies who had been jointly assessed until 2017.

**Table 1:** Description of the companies participating in the study.

Interviewee:			Interview form:		Experience with the EAC MRH:	
Type of organization	Generic drug- or research based pharmaceutical company?	Number of interviewees	Individual interview	Group interview	Joint Assessment	Joint GMP inspection
Local manufacturer	Generic drug company	1	x			x
Local manufacturer	Generic drug company	2	x			
Local manufacturer	Generic drug company	1	x		x	x
Local manufacturer	Generic drug company	2	x			
Local manufacturer	Generic drug company	2	x		x	
Local manufacturer	Generic drug company	2	x			
Local manufacturer	Generic drug company	1	x			
Local manufacturer	Generic drug company	1	x		x	x
Multinational pharmaceutical company	Research based company	3	x	x		
Multinational pharmaceutical company	Research based company	2	x	x	x	
Multinational pharmaceutical company	Research based company	3	x	x		
Multinational pharmaceutical company	Research based company	4	x			
Multinational pharmaceutical company	Generic drug company	1	x		x	
Multinational pharmaceutical company	Research based company	1		x		
Multinational pharmaceutical company	Research based company	1		x	x	
Multinational pharmaceutical company	Research based company	2		x		
Multinational pharmaceutical company	Research based company	1		x	x	
Multinational pharmaceutical company	Generic drug company	1	x			
NMRA, EAC	NA	1	x		x (implementer)	x (implementer)
NMRA, EAC	NA	2	x		x (implementer)	x (implementer)
Pharmaceutical association	NA	1		x	NA	NA
Pharmaceutical association	NA	2		x	NA	NA
**TOTAL**:		**37**	16	**9**	**9 (7 pharmaceutical companies)**	**5 (3 pharmaceutical companies)**

NA = Not Applicable

Despite repeated attempts to interview the three NMRAs and the EAC Secretariat, we were only successful in interviewing two NMRAs.

The authors LSD and CÅ participated in the majority of the interviews, including taking notes and comparison of the notes immediately after each interview had taken place. WDO participated in face-to-face interview collection and transcription with selected participants from Uganda. In most cases, the finalized notes were sent back to the participants for validation. If only one of the authors participated in the interview, the interviews were recorded and transcribed.

### Analysis

The data were analysed using thematic analysis. First, the interview transcripts/notes were reviewed. Any uncertainties in the text material were clarified with the interviewee(s). The data were then organized into the codes/categories that had emerged during the review, i.e. text elements that had connection with each other were categorized together. We then reviewed and interpreted the text within each code/category. This enabled us to describe themes/common patterns across our data set, presented in the results section of this article.

## Results

### EAC joint assessment procedure product applications

As of December 2017, two years after the introduction, 38 applications for EAC joint assessment, out of 41 applications received, were evaluated. (Three applications were reviewed subsequently in 2018). This resulted in the registration of eight products (seven patented medicines and one generic) [17–19]. Table 2 lists the medicines that were jointly evaluated in the first five joint assessments (Oct 2015-April 2017). This list includes 34 of the 38 products that were jointly evaluated until the end of 2017, and includes all of the eight products that gained registration status as per December 2017. Comparatively, TFDA received more than 2,000 applications for national registration within a three-year period (2007–2009), of which circa 65% were approved for registration [20]. The Zazibona Medicines Registration initiative (collaboration between NMRAs in Botswana, Namibia, Zambia, and Zimbabwe) evaluated 152 applications in 13 meetings from October 2013-November 2016, of which 85 had been finalized (approved, rejected or withdrawn) [21].

**Table 2:** Medicines evaluated in EAC joint assessments Oct 2015-April 2017.

Application received	Brand name	Active ingredient(s)	Strength	Dosage form	Therapeutic category	EML^1^	Generic or original drug	Applicant	EAC Join Assessment (JA) Session	Status^2^	Registration time^2^
21.07.2015	Avastin	bevacizumab	100 mg	Injection	Anti-cancer	Yes	Original drug	F.Hoffmann La Roche Limited	1^st^ EAC JA (Oct 2015)	Registered	4 months
21.07.2015	Avastin	bevacizumab	400 mg	Injection	Anti-cancer	Yes	Original drug	F.Hoffmann La Roche Limited	1^st^ EAC JA (Oct 2015)	Registered	4 months
21.07.2015	Herceptin	trastuzumab	150 mg	Injection	Anti-cancer	Yes	Original drug	F.Hoffmann La Roche Limited	1^st^ EAC JA (Oct 2015))	Registered	4 months
21.07.2015	Herceptin	trastuzumab	440 mg	Injection	Anti-cancer	Yes	Original drug	F.Hoffmann La Roche Limited	1^st^ EAC JA (Oct 2015))	Registered	4 months
11.09.2015	Vaclovir	valaciclovir	500 mg	Tablet	Anti-viral	No	Generic drug	Mylan Pharmaceutical Pvt Ltd	1^st^ EAC JA (Oct 2015)	Queried	
11.09.2015	Cardisar HT	telmisartan and diuretic	160+12.5 mg	Tablet	Cardiovascular	No	Generic drug	Mylan Pharmaceutical Pvt Ltd	1^st^ EAC JA (Oct 2015)	Queried	
11.09.2015	Cardisar HT	telmisartan and diuretic	160+25 mg	Tablet	Cardiovascular	No	Generic drug	Mylan Pharmaceutical Pvt Ltd	1^st^ EAC JA (Oct 2015)	Queried	
11.09.2015	Cardisar HT	telmisartan and diuretic	80+12.5 mg	Tablet	Cardiovascular	No	Generic drug	Mylan Pharmaceutical Pvt Ltd	1^st^ EAC JA (Oct 2015)	Queried	
22.10.2015	Abacavir Sulfate and Lamivudine	abacavir and lamivudine	60+30 mg	Tablet, dispersible	Anti-viral	Yes	Generic drug	Mylan Laboratories Limited	2^nd^ EAC JA (May 2016)	Queried	
15.01.2016	Nexavar	sorafenib	200 mg	Tablet	Anti-cancer	No	Original drug	Bayer East Africa Limited, Nairobi	2^nd^ EAC JA (May 2016)	Registered	5 months
27.01.2016	Metformin Sandoz	metformin	1000 mg	Tablet	Anti-diabetic	No^3^	Generic drug	Sandoz GmbH Kundl	2^nd^ EAC JA (May 2016)	Queried	
27.01.2016	Metformin Sandoz	metformin	500 mg	Tablet	Anti-diabetic	Yes	Generic drug	Sandoz GmbH Kundl	2^nd^ EAC JA (May 2016)	Queried	
27.01.2016	Tamoxifen	tamoxifen	20 mg	Tablet	Anti-estrogen	Yes	Generic drug	Sandoz GmbH Kundl	2^nd^ EAC JA (May 2016)	Queried	
27.01.2016	Amlodipine	amlodipine	10 mg	Tablet	Cardiovascular	No^3^	Generic drug	Sandoz GmbH Kundl	2^nd^ EAC JA (May 2016)	Queried	
27.01.2016	Amlodipine	amlodipine	5 mg	Tablet	Cardiovascular	Yes	Generic drug	Sandoz GmbH Kundl	2^nd^ EAC JA (May 2016)	Queried	
09.02.2016	Sirturo	bedaquiline	100 mg	Tablet	Anti-tuberculosis	Yes	Original drug	Janssen-Cilag International NV	3^rd^ JA (8–12 Aug 2016)	Registered	7 months
06.04.2016	Anatrozole Sandoz	anastrozole	1 mg	Tablet	Anti-cancer	Yes	Generic drug	Sandoz GmbH Kundl	3^rd^ JA (8–12 Aug 2016)	Queried	
06.04.2016	Bisoprolol	bisoprolol	2.5 mg	Tablet	Cardiovascular	No^3^	Generic drug	Sandoz GmbH Kundl	3^rd^ JA (8–12 Aug 2016)	Queried	
06.04.2016	Bisoprolol	bisoprolol	5 mg	Tablet	Cardiovascular	Yes	Generic drug	Sandoz GmbH Kundl	3^rd^ JA (8–12 Aug 2016)	Queried	
06.04.2016	Bisoprolol	bisoprolol	10 mg	Tablet	Cardiovascular	No^3^	Generic drug	Sandoz GmbH Kundl	3^rd^ JA (8–12 Aug 2016)	Queried	
06.04.2016	Furosemide	furosemide	40 mg	Tablet	Cardiovascular	Yes	Generic drug	Sandoz GmbH Kundl	3^rd^ JA (8–12 Aug 2016)	Queried	
20.04.2016	Ec-Pram	escitalopram	10 mg	Tablet	Antidepressant	No	Generic drug	Hetero Labs Limited	3^rd^ JA (8–12 Aug 2016)	Queried	
20.04.2016	Prega	pregabalin	150 mg	Tablet	Antiepileptic	No	Generic drug	Hetero Labs Limited	3^rd^ JA (8–12 Aug 2016)	Queried	
20.04.2016	Prega	pregabalin	75 mg	Tablet	Antiepileptic	No	Generic drug	Hetero Labs Limited	3^rd^ JA (8–12 Aug 2016)	Queried	
18.05.2016	Simvastatin	simvastatin	40 mg	Tablet	Cardiovascular	Yes	Generic drug	Sandoz GmbH Kundl	3^rd^ JA (8–12 Aug 2016)	Queried	
18.05.2016	Hydrochlorothiazide	hydrochlorothiazide	25 mg	Tablet	Cardiovascular	Yes	Generic drug	Sandoz GmbH Kundl	3^rd^ JA (8–12 Aug 2016)	Queried	
18.05.2016	Simvastatin	simvastatin	20 mg	Tablet	Cardiovascular	Yes	Generic drug	Sandoz GmbH Kundl	3^rd^ JA (8–12 Aug 2016)	Queried	
15.07.2016	Erbitux	cetuximab	5 mg/ml	Solution for infusion	Anti-cancer	No	Original drug	Merck (Pty) Limited, South Africa	4^th^ EAC JA (Dec 2016)	Registered	6 months
29.07.2016	Abpara	paracetamol	1%	Solution for infusion	Analgesic	No^4^	Generic drug	Abacus Parenteral Drugs Ltd (APDL), Uganda	4^th^ EAC JA (Dec 2016)	Queried	
29.07.2016	Ablevox	levofloxacin	unknown	Solution for infusion	Antibacterial	No^4^	Generic drug	Abacus Parenteral Drugs Ltd (APDL), Uganda	4^th^ EAC JA (Dec 2016)	Queried	
17.10.2016	Chlorhexidine Digluconate get	chlorhexidine	7.1%	Gel	Antiseptic	Yes	Generic drug	Universal Corporation Ltd, Kenya	4^th^ EAC JA (Dec 2016)	Queried	
08.11.2016	Kemoxyl DT	amoxicillin	250 mg	Tablet, dispersible	Antibacterial	Yes	Generic drug	Laboratory and Allied Limited, Kenya	4^th^ EAC JA (Dec 2016)	Registered	6 months
03.03.2017	Mycamine	micafungin	100 mg corresponding to 101.73 mg as sodium salt	Powder for solution for infusion	Antimycotic	No	Original drug	Astellas Pharma (Pty) Ltd, South Africa	5^th^ EAC JA (April 2017)	unknown	
03.03.2017	Mycamine	micafungin	50 mg corresponding to 50.86 mg as sodium salt	Powder for solution for infusion	Antimycotic	No	Original drug	Astellas Pharma (Pty) Ltd, South Africa	5^th^ EAC JA (April 2017)	unknown	

^1^ Medicine included in the World Health Organization Model List of Essential Medicines (WHO EML) for adults (August 2017).

^2^ Sources: List of medicinal products received under the EAC MRH Programme for Joint Assessment (October 2015 to April 2017), EAC, 2017 and Progress on Medicines Regulatory Harmonization in East African Community, Jane.H Mashingia, presentation at the ^3rd^ Biennial Scientific Conference Accra in November 2017.

^3^ The active substance in this dosage form is included in the WHO EML for adults (August 2017), but in different strength(s).

^4^ The active substance is included in the WHO EML for adults (August 2017), but in different dosage form(s).

Yet the intention with EAC MRC was to start with a small number of joint assessment applications and gradually increase as more experience is gained. The EAC Secretariat publishes “expression of interest lists” where applicants are invited to submit applications for marketing authorization for medicines considered high priority. The NMRAs interviewees stated that this allowed for sufficient time to set in place peer learning practices as well as secure the necessary training. It was stated that the countries did not necessarily trust the capabilities of others, so that this gradual start allowed the trust to grow. There was an expressed interest by the NMRAs that the number of applications for the joint assessment would rapidly increase now in order to reduce the workload of those performing the national assessments. Some of the pharmaceutical companies we spoke with felt that the medicines eligible for joint assessment were too narrowly defined, stating this was the sole reason why they had not yet applied for marketing authorization through the joint assessment procedure.

The jointly approved medicines (Table 2) represent three different therapeutic categories: anti-cancer (6 products), anti-tuberculosis (1 product) and antibiotics (1 product). The remaining medicines are mainly cardiovascular (12 products) and other medicines for non-communicable diseases (8 products). 17 of the listed products are included in the WHO Model List of Essential Medicines [22]. 22 products are manufactured by large multinational pharmaceutical manufacturers, and four are manufactured by local EAC companies. 25 products are generic medicines.

The seven patented medicines that have been approved through the joint assessment are all manufactured by multi-national pharmaceutical manufacturers. These medicines received marketing authorization in the United States in 1998 (both strengths of trastuzumab), 2004 (cetuximab and both strengths of bevacizumab), 2005 (sorafenib), and 2012 (bedaquiline) [23].

### Timelines of the EAC joint assessment procedure

The registration timelines for the eight products that gained registration status as of December 2017 are reported to be 4–7 months [17]. However, according to our interviewees, there are some challenges with these reported registration timelines since they represent the time when a positive outcome of the joint assessment had been received (a recommendation for registration), and not the receipt of the actual national marketing authorizations. As mentioned previously, marketing authorizations are still granted by the individual NMRAs.

We spoke with the majority of the pharmaceutical companies who received a positive joint assessment decision as of December 2017. They all reported delays in receiving the actual national marketing authorizations. They said that the result of the joint assessment is received within the 4–7 month timeline. However, when this is sent onto the NMRAs requesting the national marketing authorizations, it takes longer than the stipulated three months to receive the approval. Two companies stated that they were still waiting more than one year after the positive joint assessment for the national approvals. Other companies who have applied, but not yet received approval, stated that they received the first queries from the joint assessment after 10 months (in one case) and 14 months (in another case). This led some companies to conclude that the national regulatory approval pathway was in fact faster than the joint assessment procedure, and consequently to utilize the national pathways.

### EAC joint quality standard assessments

All interviewees except one who had participated in the joint assessment procedure reported that they received more numerous and stringent queries compared to the national registration procedures in the EAC countries. For some of these applicants, it had caused an unexpected registration delay, resulting in the decision to apply through the national procedure in parallel to the joint evaluation procedure. EAC-based companies seemed to struggle with some of the queries received in the joint assessment procedure, mainly related to demonstrating bioequivalence. According to the local manufacturers, this is currently often waived for local manufacturers in the national registration procedures. All of the EAC-based companies stated that there is no EAC-based centre capable of producing bioequivalence studies, meaning that they had to be sent abroad, usually at great expense. One NMRA representative stated that the national registration procedure would soon include a bioequivalence requirement.

According to the information published on the EAC Secretariat website, 13 joint GMP inspections had taken place, of which five had been issued a GMP certificate as of March 2017 (more recent information is not published on the EAC MRH website). Most of these companies are generic drug producers based in India or elsewhere, and have not applied for product registration through the joint assessment procedure within the same time period. A few local manufacturers had applied for a joint GMP inspection, but no multinational company.

The majority of the pharmaceutical companies we spoke with had not yet participated in an EAC joint GMP inspection. The reason for this was either that the company already had national GMP status and did not yet see the benefits of a joint GMP inspection, or the company had not yet applied for registration through the joint assessment procedure.

Some of the pharmaceutical companies perceived the joint EAC GMP inspections as not yet being fully implemented and that the process was unclear. Two of the companies that had undergone joint GMP inspections confirmed this perception. Both of these companies reported problems in getting all EAC countries to acknowledge the joint EAC GMP certificate. The perceived reason for this was that the country who did not recognize the EAC certificate did not participate in the joint inspection.

Several of our interviewees proposed to disconnect GMP inspections from registration, and instead implement a risk-based approach where the need for GMP inspections is evaluated case-by-case. The suggestion entailed a recognition of other regulatory authorities’ inspections, especially from Stringent Regulatory Authorities, i.e. members of ICH.

### Support for and understanding of the harmonization efforts

Overall most pharmaceutical companies were appreciative of the possibility of a joint assessment and supportive of the harmonization efforts. The ability to submit one dossier application for the entire EAC region was considered a welcome improvement by most interviewees, particularly those manufacturers serving multi-national markets. Several interviewees stated that they had submitted an application for joint dossier evaluation not only with the expectation of quicker market access but also with the intent to support the regional effort.

Yet, some manufacturers serving primarily the local market were concerned about the impact of the harmonization on their competitiveness. Since many of the EAC-based manufacturers produce a similar portfolio of products, they were concerned about greater local competition. Responding to this concern, Uganda introduced a 12% import tax on a select list of medicines that Ugandan manufacturers already produce. “Buy Uganda, Build Uganda” policy aims to protect local manufacturers from external competition, including from other EAC countries. All EAC-based manufacturer participants agreed that such measures were needed to protect vulnerable local industry. At the same time, most of them acknowledged that an import tax similar to the Ugandan import tax was against the EAC protocol.

All interviewees expressed surprise regarding the actual implementation of the harmonization efforts. The majority expected processes to be self-executing, meaning that decisions taken in joint evaluations were “automatically” accepted by the NMRAs and that receipt of both marketing authorization and GMP certification would be faster. However, NMRAs stated that quarterly stakeholder meetings have been held to assist local companies with the harmonization processes. The EAC-based companies tended to specialize on certain markets with a strong understanding of the requirements for regulatory approval. Some of the smaller companies were uninterested or unaware of regulatory requirements in other EAC markets.

Several of our interviewees expressed a desire for a centralized EAC agency with ultimate decision-making authority for jointly evaluated products, as well as handling all documents and payments related to the application. Some also pointed out the possibility to reduce duplication of efforts through mutual recognition of regulatory decisions and by implementing a centralized procedure. There are proposals to establish an EAC Centralised Medicines and Food Safety Agency [24], but no pharmaceutical company was aware of this. There is limited publically available information about these forthcoming plans. The NMRAs were also supportive of a centralized EAC agency, especially for the assessment of dossier applications for advanced therapy such as biotherapeutics, gene therapy and biosimilars.

The majority of the pharmaceutical companies perceived the implementation of harmonization efforts to be too slow, making it difficult to achieve the envisioned benefits of MRH. Many stated that the challenges in receiving national registration had discouraged them in submitting further applications until the procedure was simplified and more predictable.

Those EAC-based manufacturers who had not yet been through the joint assessment procedure said that they would need more information and training about the EAC joint assessment procedure before pursuing applications. They felt little training had been provided, resulting in uncertainties about the exact procedure and regulatory requirements.

The dependence of donor funding for the implementation of harmonization activities was mentioned in our interviews as posing a challenge for its financial sustainability. This was also highlighted by the NMRAs. Coupled with this is the limited availability of human resources within the regulatory authorities. Another challenge cited to weaken the EAC MRH was the limited awareness and knowledge about the harmonization efforts and its intended direction, scope and goals.

Despite these criticisms of the EAC MRH, it was agreed that several improvements in the region had been made, such as the strengthening of regulatory capacity, especially for countries with weak regulatory capacity. Moreover, the respondents said that if the current challenges were addressed, resulting in a more enabling regulatory environment, it would encourage them to pursue applications for product registration in the region.

### Target markets

One of the intentions of regional MRH is to improve access in smaller, less attractive markets, like Burundi, Rwanda, and South Sudan. Yet no interviewee, from either a large, medium, or small company mentioned that the harmonization had incentivized them to enter one of these smaller markets. Several EAC manufacturers stated that they already served the Burundian and Rwandan markets, but this was not due to harmonization efforts. Rwanda has accepted Kenyan marketing authorization without additional requirements; Zanzibar has accepted Tanzanian marketing authorization without additional requirements. Burundi, due to the state of political insecurity, had few regulatory requirements. Whereas companies appreciated that the regulatory processes were simplified, other barriers including logistics and political instability still made these markets unattractive.

### Free trade of pharmaceuticals in the EAC region

Free trade of pharmaceuticals is not yet a reality. Since the granting of marketing authorizations is still the sole responsibility of the different NMRAs, there is no common license that is valid across countries. All transport of medicines across borders require import and export licenses.

## Discussion

The data obtained from this study suggest that pharmaceutical companies are positive to the intent of the EAC MRH initiative. However, to be successful, the implementation requires significant improvements and sustained political commitment. The organizational design of the EAC MRH seems to have been chosen to minimize institutional change by utilizing the existing NMRAs and putting in place parallel processes. Whereas this is beneficial from a capacity-building perspective, it can also introduce significant delays given the number of entities that must process each marketing authorization and GMP inspection approval.

Unfortunately, due to the weaknesses of this design, the EAC MRH is at risk of losing the pharmaceutical companies’ interest to apply for joint assessments. The challenges related to the slow update on applications in the joint assessment procedure and receiving actual marketing authorization seem to discourage the companies. At the same time, applicants may be partially responsible for delays if documentation is not submitted in a timely manner. In the Zazibona project, the most common reasons for delay or rejection of product applications were that applicants did not respond to queries within the stated deadlines or that unacceptable bioequivalence data were submitted [21]. Our study found that local EAC manufacturers are also struggling to submit acceptable bioequivalence studies.

It appears that EAC countries have utilized the harmonization efforts to implement a higher level of quality control since all joint assessments require bioequivalence studies, whereas national marketing authorizations often waive these obligations. It is laudable that NMRAs are using the EAC MRH to enforce evidence requirements of quality-assured medicines. Yet without assisting local manufacturers with the provision of bioequivalence studies, the unintended effect may be to give large, international generic companies an advantage, thus making small, local companies even more vulnerable.

MRH could be considered a method to reduce the overall workload of the NMRAs while at the same time strengthening the remaining responsibilities. Indeed, this seems the intent of EAC MRH in that the individual NMRAs have unique responsibilities, e.g. Uganda for GMP inspections and Tanzania for joint dossier evaluations. Yet it seems that the NMRAs have not truly embraced this approach, as we heard repeated instances where one country would not accept a joint decision. This may have roots in the economic model, i.e. each country is funded by its fees to perform GMP assessments, review dossiers, and other functions. In most cases, companies were willing to pay these national fees to all regional countries in return for expedient recognition of a joint decision.

Our assessment did not provide evidence that MRH efforts contribute to bringing innovative medicines to the harmonized markets sooner. All but one of the patented medicines had been available in the American market for more than a decade at the time of joint assessment. The one exception (bedaquiline) requires local marketing authorization in order that funds from the Global Fund to Fight AIDS, Tuberculosis, and Malaria may be used to purchase the medicine. The other patented medicine currently undergoing the joint assessment process, micafungin, follows the same pattern (approved in the US in 2005). This may be because the EAC initially targeted generic medicines rather than medicines that are still under patents. It would be interesting to perform this check against the medicines reviewed by Zazibona to see if there is a similar pattern. Additionally, companies consistently reported that the smaller EAC markets were still unattractive, citing logistical problems regarding lack of wholesalers or local distributors as well as political instability. With these additional barriers, the cost savings of MRH have not yet improved the economic realities of doing business in these countries. However, it would be interesting to follow-up at a later date on the number of marketing authorizations and local distributors.

There was a clear desire from manufacturers for the creation of a centralized regional medical agency that could issue marketing authorizations and GMP inspection certificates. Indeed, many incorrectly believed that this was the original intent of the harmonization and then were surprised when they needed to pursue national marketing authorizations. It appears that the EAC Secretariat has also come to the conclusion that a regional agency would be beneficial, as it was reported to us that such an agency is in the planning progress. However, no manufacturer was cognizant of this progress. It seemed that general communications between the EAC Secretariat, NMRAs, and the manufacturers could be improved.

From our findings it appears that several improvements could rapidly increase pharmaceutical company participation in the EAC MRH. The EAC Secretariat should closely track national marketing authorizations and GMP assessments after a positive joint assessment to ensure that each country awards the registration within an appropriate timeframe. Open reporting of these data would add confidence to the processes. The EAC Secretariat and EAC countries should publicly declare whether they intend to create a centralized regional medical agency and if so, share its draft mandate for discussion. Local EAC-based pharmaceutical companies should work with NMRAs to put in place a clear plan for performing bioequivalence studies for all new product registrations of generic medicines.

There are a number of limitations to this study. Namely, this study represents primarily pharmaceutical companies’ perspectives; other stakeholder groups’ views are not represented. The number of interviews performed is numerically small, but it does encompass the majority of companies participating in the joint assessment. The study data was collected over one year, and therefore additional data from the early interviewees may have been missed. Lastly, the focus of this study was the EAC joint assessment procedure and the EAC joint GMP inspections. Consequently, we have not captured the progress and perceptions related to the other goals of the EAC MRH.

In conclusion, there is significant goodwill on behalf of pharmaceutical manufacturers both locally and internationally that MRH efforts can be a success. Significant progress has been made in implementing the EAC MRH, but additional efforts are needed. Currently few products are being assessed through the EAC joint assessment procedure and the timelines are too lengthy. Manufacturers report that they have lost faith in the joint assessment procedures and are instead reverting to the national registration processes. The EAC Free Trade Agreement does not facilitate free trade of medicines since import and export licenses are still required for medicines. In order to actually increase access to quality-assured medicines, continual improvements will be required to the EAC MRH as well as removing trade barriers. The NMRAs interviewed acknowledge that there have been “growing pains” but see increased capacity-building and stronger collaboration. If the EAC MRH is to be the model for the African MRH efforts, we hope that the evidence provided within this study is useful.

## Supporting information

S1 File Interview guide, pharmaceutical companies. (PDF)

S2 File Interview guide, national medicines regulatory authorities. (PDF)
