# Supplementary material for: Industrial perceptions of medicines regulatory harmonization in the East African Community
Source: PLoS One. 2019 Jun 19;14(6):e0218617. doi: 10.1371/journal.pone.0218617 (PMC6583972; doi:10.1371/journal.pone.0218617)
Supplement: S1 File — (PDF) [file pone.0218617.s001.pdf]

Interview guide for pharmaceutical companies

Date:
Country:
Company:
Position and years in the company (interviewee):

Questions

- Can you please describe what type of pharmaceutical company you represent (generic/research based, local/multinational, primary/secondary/tertiary production) and the company's main products?
- In which markets does your pharmaceutical company supply medicines?
- Does your company have products registered in the East African Community (Kenya, Tanzania, Zanzibar, Burundi, Rwanda, Uganda or South Sudan), and if so, where and which products?
- What is your experience with obtaining marketing approvals in the EAC?
- What are the determining factors related to the decision to apply for marketing authorization in East Africa?
- Does your company have experience with drug registration in East Africa?
- Can you please explain which procedure your company has used to obtain marketing authorization for products in East Africa (national authorization procedure, joint assessment procedure, or the WHO collaborative procedure)?
- What was your experience with obtaining marketing approval in East Africa?
- What is your view on regulatory harmonization of medicine registration in general, and its impact on public health?
- How familiar are you with the East African Community's Medicines Registration Harmonization project?
- To what extent do such regulatory harmonization efforts, with the possibility to apply for marketing authorization under a joint assessment process for the whole region (and possibly reduced registration time), influence your decision to apply for marketing authorization?
- If familiar with the EAC Medicines Registration Harmonization initiative:
    - What is your view on the EAC initiative compared to other harmonization initiatives, e.g. the ZAZIBONA collaborative medicines registration process?
- If experience with the joint assessment procedure:
    - What are the main reasons for using the Joint Assessment procedure?
    - What was your experience with the Joint Assessment procedure, compared to the national procedure? (timeline, queries etc.)
    - Which improvements would you like to see?
- How familiar are you with the regional free trade agreement in the East African Community (EAC)?
- To what extent do regional free trade agreements like the EAC free trade agreement (with bigger market potential) impact your decision to apply for marketing authorization in the concerned countries and why?

For EAC based companies only:

- What are the policies and initiatives that affect local manufactures in the EAC?
- Has the EAC free trade agreement impacted local manufactures' trade opportunities with other EAC members, or your export opportunities outside the EAC market? Please explain.
