# Supplementary material for: Industrial perceptions of medicines regulatory harmonization in the East African Community
Source: PLoS One. 2019 Jun 19;14(6):e0218617. doi: 10.1371/journal.pone.0218617 (PMC6583972; doi:10.1371/journal.pone.0218617)
Supplement: S2 File — (PDF) [file pone.0218617.s002.pdf]

Interview guide National Medicines Regulatory Authorities

Date:
Country:
Company:
Position and years in the company (interviewee):

Questions

- What is your view on medicine regulatory harmonization in general (positive and negative aspects), and its impact on public health?
- What has been your/the drug authority's role in the EAC medicine regulatory harmonization initiative? Please explain.
- What is your view on the implementation of the EAC medicine regulatory harmonization initiative? (e.g. pace, progress, regulatory aspects to be harmonized)
- What is your experience with the joint assessment procedure?
- How many joint assessments have been performed in your country? About how many regulatory assessments are performed outside the joint assessment each year?
- What are the benefits and drawbacks with the joint assessment pathway compared with other registration procedures (the national procedure, the WHO collaborative procedure)? (e.g. type of queries, timelines, level of bureaucracy/work-burden, collaboration across countries, communication with applicant etc.)
- How does the national registration of medicine approved under the joint assessment work in your country? And how does this differ from other registration pathways? Please explain.
- What is your view, and possible experience, with the harmonized standards for GMP inspections?
- Does your country have any other reciprocal agreements with the EAC in regards to medicine regulatory approval? Or other countries?
- How would you like the EAC medicine regulatory harmonization initiative to evolve?
